# (1*S*,2*S*,3*R*,4*S*,5*R*,7*S*,8*S*,10*R*,13*S*)-2-Debenzoyl-10-deacetyl-2-(3-fluoro­benzo­yl)-7,10-bis­(2,2,2-trichloro­eth­oxy­carbon­yl)baccatin III ethyl acetate monosolvate monohydrate

**DOI:** 10.1107/S1600536811002790

**Published:** 2011-02-02

**Authors:** Chen Zhang, Cheng Xie, Jun Chang, Hong-Fu Lu, Xun Sun

**Affiliations:** a School of Pharmacy, Fudan University, 826 Zhangheng Road, Shanghai 201203, People’s Republic of China

## Abstract

In the title compound, C_35_H_37_Cl_6_FO_14_·C_4_H_8_O_2_·H_2_O, the absolute configurations (1*S*,2*S*,3*R*,4*S*,5*R*,7*S*,8*S*,10*R*,13*S*) for the nine chiral centres of the mol­ecule has been determined. In the crystal, mol­ecules are linked by O—H⋯O and O—H⋯Cl hydrogen bonds.

## Related literature

For the preparation of the title compound, an inter­mediate from the synthesis of a fluorinated docetaxel analog, see: Lu *et al.* (2009 ▶). For the absolute configuration of the title compound, see: Kingston *et al.* (1982 ▶). 
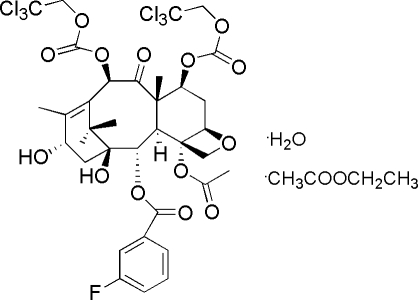


## Experimental

### 

#### Crystal data

C_35_H_37_Cl_6_FO_14_·C_4_H_8_O_2_·H_2_O
*M* *_r_* = 1019.47
Orthorhombic, 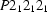

*a* = 14.7037 (11) Å
*b* = 16.6601 (12) Å
*c* = 18.9258 (14) Å
*V* = 4636.2 (6) Å^3^
*Z* = 4
Mo *K*α radiation
μ = 0.44 mm^−1^
*T* = 293 K
0.40 × 0.31 × 0.29 mm

#### Data collection

Bruker SMART CCD area-detector diffractometer
Absorption correction: multi-scan (*SADABS*; Bruker, 2001 ▶) *T*_min_ = 0.604, *T*_max_ = 1.000
25522 measured reflections
9094 independent reflections
6096 reflections with *I* > 2σ(*I*)
*R*_int_ = 0.053

#### Refinement

*R*[*F* ^2^ > 2σ(*F* ^2^)] = 0.051
*wR*(*F* ^2^) = 0.115
*S* = 0.91
9094 reflections
628 parameters
101 restraints
H atoms treated by a mixture of independent and constrained refinement
Δρ_max_ = 0.64 e Å^−3^
Δρ_min_ = −0.32 e Å^−3^
Absolute structure: Flack (1983 ▶), 4052 Friedel pairs
Flack parameter: 0.03 (6)

### 

Data collection: *SMART* (Bruker, 2001 ▶); cell refinement: *SAINT* (Bruker, 2001 ▶); data reduction: *SAINT*; program(s) used to solve structure: *SHELXS97* (Sheldrick, 2008 ▶); program(s) used to refine structure: *SHELXL97* (Sheldrick, 2008 ▶); molecular graphics: *SHELXTL* (Sheldrick, 2008 ▶); software used to prepare material for publication: *SHELXTL*.

## Supplementary Material

Crystal structure: contains datablocks I, global. DOI: 10.1107/S1600536811002790/zs2080sup1.cif

Structure factors: contains datablocks I. DOI: 10.1107/S1600536811002790/zs2080Isup2.hkl

Additional supplementary materials:  crystallographic information; 3D view; checkCIF report

## Figures and Tables

**Table 1:** Hydrogen-bond geometry (Å, °)

*D*—H⋯*A*	*D*—H	H⋯*A*	*D*⋯*A*	*D*—H⋯*A*
O17—H17*E*⋯Cl1^i^	0.89 (2)	2.85 (6)	3.582 (5)	141 (6)
O17—H17*E*⋯O9^i^	0.89 (2)	2.48 (7)	3.241 (6)	143 (9)
O14—H14⋯O17	0.83 (2)	2.00 (2)	2.791 (6)	161 (4)
O1—H1⋯O15	0.82 (2)	2.01 (3)	2.786 (6)	158 (5)
O17—H17*D*⋯O1^ii^	0.91 (2)	2.25 (4)	3.085 (6)	152 (6)

Symmetry codes: (i) 
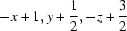
; (ii) 
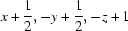
.

**Fig. 1.**
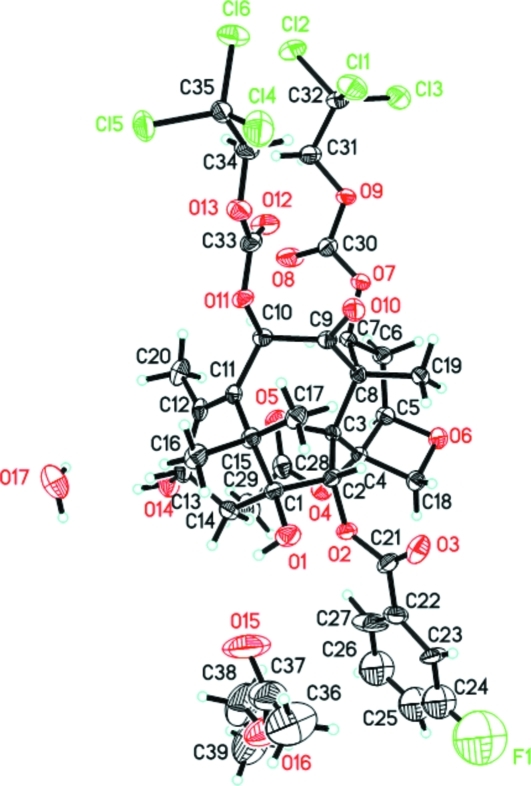
Molecular configuration and atom numbering scheme for (I) with probaility ellipsoids drawn at the 40% probability level???.

**Fig. 2.**
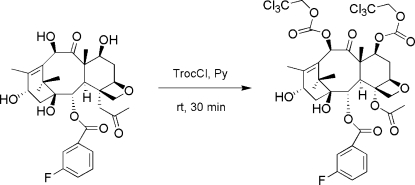
Reaction scheme for the synthesis of (I)

## supplementary crystallographic information

### Comment

In our research on the synthesis of a series of novel fluorinated docetaxel analogs, one of the key intermediate products, the title compound 7,10-di- (2,2,2-trichloroethyloxycarbonyl)-2-debenzoyl- 2-(3-fluorobenzoyl)-10-deacetylbaccatin III monohydrate ethyl acetate monosolvate, C_35_H_37_Cl_6_FO_14_. C_4_H_8_O_2_. H_2_O (I) (Fig. 1) was obtained from 10-deacetylbaccatin III (Kingston *et al.*,1982). The reaction scheme (Lu *et al.*, 2009) is shown in Fig.2. Absolute configuration(1*S*,2S,3*R*,4S,5*R*,7S,8*S*,10*R*,13*S*) for the nine chiral centres of the molecule has been determined. In the crystal structure, molecules are linked by hydroxy and water O—H···O hydrogen bonds (Table 1).

### Experimental

To a solution of 2-debenzoyl-2-(*m*-fluorobenzoyl)-10-deacetylbaccatin III (1.03 g, 1.84 mmol) in anhydrous pyridine (20 ml) was added 2,2,2-trichloroethylchloroformate (0.85 ml, 6.17 mmol) dropwise at 273 K. The reaction mixture was then warmed to room temperature and further stirred for 30 min. The reaction mixture was then quenched with water and the solvent was removed under reduced pressure. The residue was dissolved in DCM, and washed with dilute HCl and brine. The organic layer was dried with anhydrous Na_2_SO_4_, filtered, and concentrated *in vacuo*. The crude residue was purified by flash column chromatography (petroleum ether/ ethyl acetate 2/1) to give the title compound (1.58 g, 94% yield) as a white solid. Suitable crystals were obtained by recrystallization from hexane and DCM (m.p. 495–497 K). ^1^H NMR (300 MHz, CDCl_3_): d 1.12 (s, 3H), 1.15 (s, 3H), 1.85 (s, 3H), 2.17 (s, 3H), 2.30 (m, 2H), 2.31 (s, 3H), 2.05 and 2.65 (2 m, 2H), 3.98 (d, 1H, J = 6.6 Hz), 4.14 and 4.33 (2 d, 2H, J = 8.4 Hz), 4.61 and 4.92 (2 d, 2H, J=12.0 Hz), 4.78 (d, 2H, J = 12.0 Hz), 4.90 (m, 1H), 5.00 (d, 1H, J = 7.8 Hz), 5.59 (m, 1H), 5.62 (d, 1H, J = 7.5 Hz), 6.27 (s, 1H), 7.33 (m, 1H), 7.48 (m, 1H), 7.79 (m, 1H), 7.90 (d, 1H, J = 7.5 Hz); ^13^C NMR (100 MHz, CDCl3): d 10.6, 15.4, 20.1, 22.5, 26.6, 33.3, 38.4, 42.6, 47.4, 56.3, 67.8, 74.6, 76.2, 76.6, 77.1, 77.4, 78.7, 79.7, 80.4, 83.7, 94.2, 94.3, 116.9, 120.9, 125.9, 130.4, 130.8, 131.4, 146.6, 153.2, 153.3, 162.6 (d, J_C—F_ = 246.4 Hz), 165.7, 170.8, 201.1; ESIMS m/*z* 933.0 [*M* + Na^+^]; HRMS (MALDI) m/*z* calcd for C_35_H_37_Cl_6_FO_14_Na^+^ [*M* + Na^+^]: 933.0215, found: 933.01908.

### Refinement

Hydrogen atoms of the hydroxy groups and the water molecule were located by difference methods and both positional and isotropic displacement parameters were refined. Other H atoms were positioned geometrically and treated as riding with C—H = 0.96–0.98 Å and *U*_iso_ = 1.2 or 1.5*U*eq(C). The F atom was significantly disordered and was subsequently refined isotropically. The absolute configuration for the nine chiral centres in the molecule have been assigned C1(S), C2(S), C3(*R*), C4(S), C5(*R*), C7(S), C8(S), C10(*R*), C13(S) on the basis of the Flack structure parameter [0.03 (6)] (Flack,1983) (atom mames are those arbitrarily assigned in this crystallographic study).

### Crystal data

**Table:**

C_35_H_37_Cl_6_FO_14_·C_4_H_8_O_2_·H_2_O	*D*_x_ = 1.461 Mg m^−^^3^
*M**_r_* = 1019.47	Melting point = 495–497 K
Orthorhombic, *P*2_1_2_1_2_1_	Mo *K*α radiation, λ = 0.71073 Å
Hall symbol: P 2ac 2ab	Cell parameters from 4411 reflections
*a* = 14.7037 (11) Å	θ = 5.0–38.3°
*b* = 16.6601 (12) Å	µ = 0.44 mm^−^^1^
*c* = 18.9258 (14) Å	*T* = 293 K
*V* = 4636.2 (6) Å^3^	Prismatic, colorless
*Z* = 4	0.40 × 0.31 × 0.29 mm
*F*(000) = 2112	

### Data collection

**Table:**

Bruker SMART CCD area-detector diffractometer	9094 independent reflections
Radiation source: fine-focus sealed tube	6096 reflections with *I* > 2σ(*I*)
graphite	*R*_int_ = 0.053
φ and ω scans	θ_max_ = 26.0°, θ_min_ = 1.6°
Absorption correction: multi-scan (*SADABS*; Bruker, 2001)	*h* = −13→18
*T*_min_ = 0.604, *T*_max_ = 1.000	*k* = −19→20
25522 measured reflections	*l* = −22→23

### Refinement

**Table:**

Refinement on *F*^2^	Secondary atom site location: difference Fourier map
Least-squares matrix: full	Hydrogen site location: inferred from neighbouring sites
*R*[*F*^2^ > 2σ(*F*^2^)] = 0.051	H atoms treated by a mixture of independent and constrained refinement
*wR*(*F*^2^) = 0.115	*w* = 1/[σ^2^(*F*_o_^2^) + (0.0513*P*)^2^] where *P* = (*F*_o_^2^ + 2*F*_c_^2^)/3
*S* = 0.91	(Δ/σ)_max_ = 0.013
9094 reflections	Δρ_max_ = 0.64 e Å^−^^3^
628 parameters	Δρ_min_ = −0.32 e Å^−^^3^
101 restraints	Absolute structure: Flack (1983), 4052 Friedel pairs
Primary atom site location: structure-invariant direct methods	Flack parameter: 0.03 (6)

### Special details

**Table:**

Geometry. All e.s.d.'s (except the e.s.d. in the dihedral angle between two l.s. planes) are estimated using the full covariance matrix. The cell e.s.d.'s are taken into account individually in the estimation of e.s.d.'s in distances, angles and torsion angles; correlations between e.s.d.'s in cell parameters are only used when they are defined by crystal symmetry. An approximate (isotropic) treatment of cell e.s.d.'s is used for estimating e.s.d.'s involving l.s. planes.
Refinement. Refinement of *F*^2^ against ALL reflections. The weighted *R*-factor *wR* and goodness of fit *S* are based on *F*^2^, conventional *R*-factors *R* are based on *F*, with *F* set to zero for negative *F*^2^. The threshold expression of *F*^2^ > σ(*F*^2^) is used only for calculating *R*-factors(gt) *etc*. and is not relevant to the choice of reflections for refinement. *R*-factors based on *F*^2^ are statistically about twice as large as those based on *F*, and *R*- factors based on ALL data will be even larger.

### Fractional atomic coordinates and isotropic or equivalent isotropic displacement parameters (Å^2^)

**Table:**

	*x*	*y*	*z*	*U*_iso_*/*U*_eq_	Occ. (<1)
Cl1	0.32716 (9)	0.01873 (8)	1.04792 (7)	0.0699 (4)	
Cl2	0.48384 (11)	0.04791 (9)	1.13542 (6)	0.0841 (5)	
Cl3	0.45947 (9)	−0.10584 (7)	1.06832 (6)	0.0652 (4)	
Cl4	0.08093 (10)	0.25135 (9)	0.93518 (9)	0.0931 (5)	
Cl5	0.21962 (10)	0.36674 (7)	0.96872 (8)	0.0782 (5)	
Cl6	0.17098 (13)	0.24496 (9)	1.06934 (8)	0.1047 (6)	
O1	0.3735 (2)	0.16138 (19)	0.49241 (15)	0.0514 (8)	
O2	0.48084 (18)	0.03348 (15)	0.50337 (12)	0.0380 (6)	
O3	0.3511 (2)	−0.02415 (19)	0.46551 (17)	0.0642 (9)	
O4	0.64110 (17)	0.00931 (15)	0.57227 (13)	0.0385 (6)	
O5	0.7005 (2)	0.05200 (18)	0.67458 (16)	0.0538 (8)	
O6	0.5595 (2)	−0.15701 (15)	0.62706 (14)	0.0488 (8)	
O7	0.47088 (17)	−0.02112 (15)	0.82322 (12)	0.0365 (6)	
O8	0.5838 (2)	0.0466 (2)	0.87704 (15)	0.0664 (10)	
O9	0.46637 (18)	−0.00885 (15)	0.93540 (12)	0.0408 (7)	
O10	0.30922 (18)	0.05437 (17)	0.75999 (15)	0.0470 (7)	
O11	0.34759 (18)	0.20512 (15)	0.78236 (12)	0.0378 (6)	
O12	0.3935 (2)	0.14650 (18)	0.88410 (14)	0.0578 (9)	
O13	0.2698 (2)	0.22498 (16)	0.87740 (14)	0.0498 (8)	
O14	0.6592 (2)	0.2433 (2)	0.59204 (18)	0.0644 (9)	
O15	0.4525 (4)	0.2095 (4)	0.3655 (2)	0.163 (2)	
O16	0.5049 (3)	0.1760 (3)	0.2632 (2)	0.1125 (16)	
O17	0.7215 (3)	0.4008 (3)	0.6057 (3)	0.0948 (13)	
C1	0.4306 (3)	0.1568 (2)	0.55338 (18)	0.0352 (9)	
C2	0.4391 (3)	0.0639 (2)	0.56755 (18)	0.0338 (9)	
H2	0.3779	0.0411	0.5718	0.041*	
C3	0.4974 (2)	0.0350 (2)	0.63144 (17)	0.0275 (8)	
H3	0.5330	0.0818	0.6464	0.033*	
C4	0.5681 (3)	−0.0301 (2)	0.61001 (18)	0.0309 (8)	
C5	0.6021 (3)	−0.0914 (2)	0.66434 (18)	0.0356 (9)	
H5	0.6684	−0.0962	0.6615	0.043*	
C6	0.5727 (3)	−0.0846 (2)	0.74108 (19)	0.0396 (10)	
H6A	0.6263	−0.0832	0.7709	0.047*	
H6B	0.5377	−0.1318	0.7538	0.047*	
C7	0.5157 (3)	−0.0099 (2)	0.75516 (17)	0.0312 (8)	
H7	0.5562	0.0367	0.7583	0.037*	
C8	0.4412 (2)	0.0076 (2)	0.69925 (18)	0.0290 (8)	
C9	0.3801 (3)	0.0733 (2)	0.73402 (18)	0.0304 (8)	
C10	0.4163 (3)	0.1582 (2)	0.74474 (18)	0.0314 (9)	
H10	0.4702	0.1547	0.7750	0.038*	
C11	0.4436 (3)	0.1997 (2)	0.67764 (18)	0.0325 (9)	
C12	0.5294 (3)	0.2261 (2)	0.6700 (2)	0.0373 (9)	
C13	0.5632 (3)	0.2488 (2)	0.5977 (2)	0.0455 (11)	
H13	0.5451	0.3043	0.5881	0.055*	
C14	0.5237 (3)	0.1953 (2)	0.54023 (19)	0.0460 (11)	
H14A	0.5669	0.1525	0.5313	0.055*	
H14B	0.5194	0.2268	0.4973	0.055*	
C15	0.3795 (3)	0.2012 (2)	0.61382 (19)	0.0356 (9)	
C16	0.3599 (3)	0.2890 (2)	0.5920 (2)	0.0498 (11)	
H16A	0.3268	0.3154	0.6289	0.075*	
H16B	0.3246	0.2895	0.5493	0.075*	
H16C	0.4164	0.3165	0.5841	0.075*	
C17	0.2842 (3)	0.1642 (2)	0.6257 (2)	0.0449 (10)	
H17A	0.2906	0.1090	0.6395	0.067*	
H17B	0.2499	0.1672	0.5826	0.067*	
H17C	0.2532	0.1933	0.6622	0.067*	
C18	0.5361 (3)	−0.1049 (2)	0.5696 (2)	0.0400 (10)	
H18A	0.4715	−0.1045	0.5592	0.048*	
H18B	0.5710	−0.1153	0.5271	0.048*	
C19	0.3806 (3)	−0.0650 (2)	0.6853 (2)	0.0377 (9)	
H19A	0.4176	−0.1099	0.6717	0.057*	
H19B	0.3386	−0.0527	0.6480	0.057*	
H19C	0.3474	−0.0781	0.7274	0.057*	
C20	0.5979 (3)	0.2312 (3)	0.7288 (2)	0.0522 (12)	
H20A	0.5668	0.2361	0.7732	0.078*	
H20B	0.6361	0.2772	0.7217	0.078*	
H20C	0.6345	0.1835	0.7291	0.078*	
C21	0.4298 (4)	−0.0068 (2)	0.4567 (2)	0.0463 (11)	
F1	0.4305 (8)	−0.1272 (5)	0.2284 (3)	0.198 (3)	0.70
C22	0.4730 (5)	−0.0350 (5)	0.3940 (3)	0.026 (2)	0.526 (16)
C23	0.4227 (6)	−0.0638 (5)	0.3372 (3)	0.041 (3)	0.526 (16)
H23	0.3595	−0.0655	0.3398	0.049*	0.526 (16)
C24	0.4667 (8)	−0.0900 (5)	0.2764 (3)	0.064 (3)	0.526 (16)
C25	0.5611 (8)	−0.0874 (5)	0.2725 (3)	0.083 (5)	0.526 (16)
H25	0.5906	−0.1049	0.2319	0.099*	0.526 (16)
C26	0.6114 (6)	−0.0587 (6)	0.3293 (4)	0.073 (4)	0.526 (16)
H26	0.6745	−0.0570	0.3267	0.087*	0.526 (16)
C27	0.5673 (5)	−0.0325 (5)	0.3901 (3)	0.042 (3)	0.526 (16)
H27	0.6010	−0.0133	0.4281	0.050*	0.526 (16)
F1'	0.5191 (10)	−0.1369 (6)	0.2353 (4)	0.197 (9)	0.30
C22'	0.5049 (9)	−0.0223 (7)	0.3965 (5)	0.061 (4)	0.474 (16)
C23'	0.4661 (9)	−0.0641 (7)	0.3405 (6)	0.081 (5)	0.474 (16)
H23'	0.4035	−0.0716	0.3388	0.097*	0.474 (16)
C24'	0.5210 (11)	−0.0946 (6)	0.2871 (5)	0.066 (4)	0.474 (16)
C25'	0.6146 (10)	−0.0835 (6)	0.2897 (5)	0.093 (5)	0.474 (16)
H25'	0.6513	−0.1039	0.2540	0.112*	0.474 (16)
C26'	0.6534 (8)	−0.0417 (7)	0.3457 (6)	0.112 (6)	0.474 (16)
H26'	0.7160	−0.0342	0.3475	0.135*	0.474 (16)
C27'	0.5985 (9)	−0.0112 (8)	0.3992 (5)	0.083 (5)	0.474 (16)
H27'	0.6244	0.0168	0.4366	0.100*	0.474 (16)
C28	0.7039 (3)	0.0475 (2)	0.6126 (3)	0.0469 (11)	
C29	0.7772 (3)	0.0835 (3)	0.5674 (3)	0.0630 (13)	
H29A	0.7614	0.1378	0.5557	0.094*	
H29B	0.7833	0.0526	0.5248	0.094*	
H29C	0.8338	0.0830	0.5927	0.094*	
C30	0.5148 (3)	0.0097 (2)	0.8779 (2)	0.0387 (9)	
C31	0.4976 (3)	0.0306 (3)	0.99812 (19)	0.0468 (11)	
H31A	0.4888	0.0881	0.9940	0.056*	
H31B	0.5619	0.0203	1.0050	0.056*	
C32	0.4442 (3)	−0.0013 (2)	1.0595 (2)	0.0462 (11)	
C33	0.3432 (3)	0.1873 (2)	0.8512 (2)	0.0440 (11)	
C34	0.2589 (4)	0.2100 (3)	0.9515 (2)	0.0605 (14)	
H34A	0.3157	0.2198	0.9761	0.073*	
H34B	0.2414	0.1545	0.9592	0.073*	
C35	0.1856 (4)	0.2657 (3)	0.9791 (2)	0.0627 (14)	
C36	0.3518 (5)	0.1838 (7)	0.2640 (5)	0.167 (4)	
H36A	0.3408	0.1283	0.2535	0.250*	
H36B	0.3562	0.2137	0.2208	0.250*	
H36C	0.3025	0.2045	0.2919	0.250*	
C37	0.4357 (7)	0.1913 (5)	0.3031 (4)	0.129 (3)	
C38	0.5947 (6)	0.1783 (5)	0.2986 (4)	0.137 (3)	
H38A	0.6089	0.2330	0.3124	0.164*	
H38B	0.5931	0.1455	0.3409	0.164*	
C39	0.6630 (5)	0.1490 (6)	0.2517 (5)	0.147 (3)	
H39A	0.6456	0.0970	0.2345	0.220*	
H39B	0.7198	0.1449	0.2764	0.220*	
H39C	0.6695	0.1853	0.2126	0.220*	
H1	0.391 (4)	0.187 (3)	0.4583 (18)	0.08 (2)*	
H14	0.689 (3)	0.2851 (17)	0.593 (2)	0.050 (14)*	
H17D	0.749 (4)	0.376 (4)	0.569 (2)	0.13 (3)*	
H17E	0.690 (7)	0.440 (5)	0.585 (4)	0.21 (7)*	

### Atomic displacement parameters (Å^2^)

**Table:**

	*U*^11^	*U*^22^	*U*^33^	*U*^12^	*U*^13^	*U*^23^
Cl1	0.0532 (7)	0.0841 (9)	0.0724 (8)	0.0076 (7)	0.0175 (7)	0.0100 (7)
Cl2	0.1221 (13)	0.0941 (10)	0.0360 (6)	−0.0281 (9)	−0.0002 (7)	−0.0152 (6)
Cl3	0.0857 (10)	0.0550 (7)	0.0548 (7)	0.0017 (7)	0.0015 (7)	0.0104 (6)
Cl4	0.0709 (10)	0.0803 (10)	0.1283 (13)	−0.0074 (8)	0.0329 (10)	−0.0173 (10)
Cl5	0.0888 (10)	0.0376 (6)	0.1083 (11)	−0.0080 (7)	0.0518 (9)	−0.0079 (7)
Cl6	0.1549 (16)	0.0884 (11)	0.0707 (9)	−0.0151 (10)	0.0685 (10)	−0.0060 (8)
O1	0.066 (2)	0.0535 (19)	0.0343 (17)	0.0101 (17)	−0.0127 (16)	0.0037 (15)
O2	0.0474 (17)	0.0383 (15)	0.0284 (13)	0.0033 (13)	0.0035 (13)	−0.0035 (11)
O3	0.065 (2)	0.061 (2)	0.066 (2)	−0.0042 (19)	−0.0187 (19)	−0.0175 (17)
O4	0.0352 (15)	0.0419 (15)	0.0383 (14)	0.0036 (13)	0.0098 (13)	0.0014 (13)
O5	0.0481 (19)	0.062 (2)	0.0513 (19)	−0.0068 (16)	−0.0071 (15)	−0.0079 (17)
O6	0.070 (2)	0.0274 (14)	0.0491 (16)	0.0024 (14)	−0.0006 (16)	0.0016 (13)
O7	0.0407 (16)	0.0424 (15)	0.0265 (12)	−0.0062 (13)	0.0011 (12)	0.0014 (12)
O8	0.052 (2)	0.108 (3)	0.0388 (16)	−0.0339 (19)	0.0029 (15)	−0.0104 (18)
O9	0.0460 (17)	0.0527 (17)	0.0236 (12)	−0.0109 (14)	0.0009 (13)	0.0019 (12)
O10	0.0385 (17)	0.0447 (16)	0.0578 (18)	−0.0026 (14)	0.0187 (15)	−0.0051 (15)
O11	0.0459 (17)	0.0351 (15)	0.0325 (14)	0.0119 (13)	0.0032 (13)	−0.0053 (12)
O12	0.079 (2)	0.0545 (19)	0.0405 (16)	0.0324 (18)	0.0049 (16)	0.0056 (15)
O13	0.062 (2)	0.0432 (17)	0.0440 (16)	0.0147 (15)	0.0193 (16)	0.0019 (14)
O14	0.055 (2)	0.056 (2)	0.082 (2)	−0.0113 (18)	0.0204 (18)	−0.0080 (19)
O15	0.196 (5)	0.232 (6)	0.061 (3)	−0.054 (5)	0.022 (3)	−0.007 (3)
O16	0.079 (3)	0.176 (5)	0.082 (3)	−0.019 (3)	0.013 (3)	−0.039 (3)
O17	0.072 (3)	0.077 (3)	0.136 (4)	−0.010 (2)	0.009 (3)	0.024 (3)
C1	0.045 (2)	0.034 (2)	0.0270 (19)	0.0055 (19)	−0.0045 (18)	0.0021 (16)
C2	0.035 (2)	0.034 (2)	0.0324 (19)	0.0007 (17)	0.0024 (18)	−0.0057 (17)
C3	0.030 (2)	0.0246 (19)	0.0283 (18)	−0.0001 (16)	0.0029 (16)	−0.0019 (15)
C4	0.038 (2)	0.027 (2)	0.0278 (18)	0.0017 (17)	0.0034 (17)	0.0009 (16)
C5	0.039 (2)	0.031 (2)	0.037 (2)	0.0076 (18)	0.0047 (19)	0.0002 (17)
C6	0.050 (3)	0.034 (2)	0.035 (2)	0.008 (2)	−0.001 (2)	0.0082 (18)
C7	0.036 (2)	0.033 (2)	0.0249 (17)	−0.0037 (17)	0.0041 (17)	0.0022 (16)
C8	0.030 (2)	0.0247 (19)	0.0321 (19)	−0.0005 (16)	0.0017 (16)	0.0025 (15)
C9	0.030 (2)	0.033 (2)	0.0282 (19)	0.0034 (17)	−0.0001 (17)	0.0001 (16)
C10	0.028 (2)	0.032 (2)	0.034 (2)	0.0094 (17)	0.0032 (17)	−0.0054 (17)
C11	0.041 (2)	0.0205 (19)	0.036 (2)	0.0052 (17)	0.0018 (19)	−0.0040 (16)
C12	0.043 (2)	0.0212 (19)	0.048 (2)	0.0007 (18)	−0.002 (2)	−0.0060 (17)
C13	0.048 (3)	0.029 (2)	0.060 (3)	−0.006 (2)	0.012 (2)	−0.001 (2)
C14	0.063 (3)	0.037 (2)	0.038 (2)	−0.003 (2)	0.009 (2)	0.0048 (19)
C15	0.041 (2)	0.029 (2)	0.037 (2)	0.0072 (18)	−0.0038 (19)	0.0001 (18)
C16	0.056 (3)	0.041 (2)	0.052 (2)	0.015 (2)	−0.009 (2)	−0.004 (2)
C17	0.045 (3)	0.042 (2)	0.048 (2)	0.005 (2)	−0.010 (2)	−0.006 (2)
C18	0.053 (3)	0.028 (2)	0.039 (2)	0.0057 (19)	0.004 (2)	−0.0037 (18)
C19	0.038 (2)	0.034 (2)	0.041 (2)	−0.0032 (19)	0.0051 (19)	0.0015 (18)
C20	0.045 (3)	0.043 (3)	0.069 (3)	−0.004 (2)	−0.007 (2)	−0.014 (2)
C21	0.071 (3)	0.038 (2)	0.030 (2)	0.004 (2)	−0.009 (2)	−0.0035 (18)
F1	0.288 (8)	0.198 (6)	0.107 (4)	−0.049 (6)	−0.005 (5)	−0.044 (5)
C22	0.055 (5)	0.014 (4)	0.008 (4)	−0.014 (4)	0.002 (3)	−0.005 (3)
C23	0.061 (6)	0.041 (5)	0.019 (4)	−0.018 (4)	−0.014 (4)	−0.004 (3)
C24	0.106 (8)	0.062 (6)	0.023 (4)	−0.015 (6)	−0.024 (5)	−0.021 (4)
C25	0.096 (10)	0.087 (8)	0.066 (7)	−0.007 (7)	0.029 (7)	−0.013 (6)
C26	0.120 (8)	0.080 (7)	0.018 (5)	0.027 (6)	0.015 (5)	−0.020 (5)
C27	0.042 (6)	0.055 (6)	0.028 (4)	0.007 (5)	0.007 (4)	−0.012 (4)
F1'	0.198 (12)	0.197 (12)	0.197 (12)	−0.005 (9)	−0.010 (9)	−0.015 (9)
C22'	0.078 (8)	0.054 (7)	0.051 (6)	−0.022 (7)	−0.004 (6)	−0.004 (5)
C23'	0.075 (8)	0.079 (8)	0.089 (8)	−0.011 (7)	0.000 (7)	0.016 (7)
C24'	0.099 (9)	0.071 (7)	0.029 (5)	−0.020 (7)	−0.010 (6)	−0.018 (5)
C25'	0.116 (9)	0.090 (8)	0.073 (8)	−0.004 (7)	0.044 (7)	−0.001 (7)
C26'	0.176 (11)	0.091 (9)	0.070 (8)	−0.017 (8)	−0.047 (8)	0.001 (7)
C27'	0.091 (9)	0.094 (9)	0.065 (7)	−0.023 (7)	0.010 (7)	−0.014 (6)
C28	0.040 (3)	0.037 (2)	0.064 (3)	0.006 (2)	0.003 (2)	−0.001 (2)
C29	0.047 (3)	0.056 (3)	0.086 (4)	0.000 (2)	0.022 (3)	0.001 (3)
C30	0.039 (2)	0.042 (2)	0.035 (2)	−0.002 (2)	−0.004 (2)	−0.0008 (19)
C31	0.047 (3)	0.057 (3)	0.037 (2)	−0.003 (2)	0.002 (2)	−0.003 (2)
C32	0.053 (3)	0.054 (3)	0.032 (2)	−0.003 (2)	0.003 (2)	−0.003 (2)
C33	0.059 (3)	0.035 (2)	0.038 (2)	0.009 (2)	0.009 (2)	−0.006 (2)
C34	0.085 (4)	0.043 (3)	0.054 (3)	0.012 (3)	0.033 (3)	0.003 (2)
C35	0.085 (4)	0.036 (3)	0.068 (3)	−0.006 (2)	0.043 (3)	−0.008 (2)
C36	0.063 (5)	0.275 (13)	0.162 (8)	−0.039 (6)	0.010 (6)	−0.044 (8)
C37	0.139 (8)	0.163 (8)	0.086 (5)	−0.033 (6)	0.034 (6)	−0.006 (5)
C38	0.127 (7)	0.123 (6)	0.161 (8)	−0.010 (6)	−0.071 (7)	−0.028 (6)
C39	0.081 (5)	0.173 (9)	0.187 (9)	−0.011 (6)	−0.012 (6)	−0.039 (7)

### Geometric parameters (Å, °)

**Table:**

Cl1—C32	1.766 (4)	C14—H14A	0.9700
Cl2—C32	1.754 (4)	C14—H14B	0.9700
Cl3—C32	1.764 (4)	C15—C17	1.546 (5)
Cl4—C35	1.765 (6)	C15—C16	1.547 (5)
Cl5—C35	1.766 (4)	C16—H16A	0.9600
Cl6—C35	1.756 (5)	C16—H16B	0.9600
O1—C1	1.429 (4)	C16—H16C	0.9600
O1—H1	0.82 (2)	C17—H17A	0.9600
O2—C21	1.340 (5)	C17—H17B	0.9600
O2—C2	1.452 (4)	C17—H17C	0.9600
O3—C21	1.203 (5)	C18—H18A	0.9700
O4—C28	1.358 (5)	C18—H18B	0.9700
O4—C4	1.447 (4)	C19—H19A	0.9600
O5—C28	1.176 (5)	C19—H19B	0.9600
O6—C18	1.433 (4)	C19—H19C	0.9600
O6—C5	1.444 (4)	C20—H20A	0.9600
O7—C30	1.323 (4)	C20—H20B	0.9600
O7—C7	1.459 (4)	C20—H20C	0.9600
O8—C30	1.187 (5)	C21—C22	1.425 (6)
O9—C30	1.337 (4)	C21—C22'	1.607 (9)
O9—C31	1.432 (4)	F1—C24	1.223 (8)
O10—C9	1.194 (4)	C22—C23	1.3900
O11—C33	1.337 (5)	C22—C27	1.3900
O11—C10	1.463 (4)	C23—C24	1.3900
O12—C33	1.182 (5)	C23—H23	0.9300
O13—C33	1.344 (5)	C24—C25	1.3900
O13—C34	1.433 (5)	C25—C26	1.3900
O14—C13	1.419 (5)	C25—H25	0.9300
O14—H14	0.83 (2)	C26—C27	1.3900
O15—C37	1.244 (8)	C26—H26	0.9300
O16—C37	1.292 (9)	C27—H27	0.9300
O16—C38	1.480 (8)	F1'—C24'	1.208 (9)
O17—H17D	0.91 (2)	C22'—C23'	1.3900
O17—H17E	0.89 (2)	C22'—C27'	1.3900
C1—C14	1.532 (6)	C23'—C24'	1.3900
C1—C15	1.556 (5)	C23'—H23'	0.9300
C1—C2	1.576 (5)	C24'—C25'	1.3900
C2—C3	1.559 (5)	C25'—C26'	1.3900
C2—H2	0.9800	C25'—H25'	0.9300
C3—C4	1.556 (5)	C26'—C27'	1.3900
C3—C8	1.594 (5)	C26'—H26'	0.9300
C3—H3	0.9800	C27'—H27'	0.9300
C4—C5	1.533 (5)	C28—C29	1.501 (6)
C4—C18	1.537 (5)	C29—H29A	0.9600
C5—C6	1.520 (5)	C29—H29B	0.9600
C5—H5	0.9800	C29—H29C	0.9600
C6—C7	1.523 (5)	C31—C32	1.499 (5)
C6—H6A	0.9700	C31—H31A	0.9700
C6—H6B	0.9700	C31—H31B	0.9700
C7—C8	1.550 (5)	C34—C35	1.516 (6)
C7—H7	0.9800	C34—H34A	0.9700
C8—C19	1.525 (5)	C34—H34B	0.9700
C8—C9	1.562 (5)	C36—C37	1.444 (10)
C9—C10	1.526 (5)	C36—H36A	0.9600
C10—C11	1.500 (5)	C36—H36B	0.9600
C10—H10	0.9800	C36—H36C	0.9600
C11—C12	1.344 (5)	C38—C39	1.427 (9)
C11—C15	1.532 (5)	C38—H38A	0.9700
C12—C20	1.504 (5)	C38—H38B	0.9700
C12—C13	1.504 (5)	C39—H39A	0.9600
C13—C14	1.522 (5)	C39—H39B	0.9600
C13—H13	0.9800	C39—H39C	0.9600
			
C1—O1—H1	119 (4)	H19A—C19—H19B	109.5
C21—O2—C2	119.3 (3)	C8—C19—H19C	109.5
C28—O4—C4	116.1 (3)	H19A—C19—H19C	109.5
C18—O6—C5	91.0 (2)	H19B—C19—H19C	109.5
C30—O7—C7	114.9 (3)	C12—C20—H20A	109.5
C30—O9—C31	113.5 (3)	C12—C20—H20B	109.5
C33—O11—C10	112.8 (3)	H20A—C20—H20B	109.5
C33—O13—C34	111.7 (3)	C12—C20—H20C	109.5
C13—O14—H14	118 (3)	H20A—C20—H20C	109.5
C37—O16—C38	115.7 (6)	H20B—C20—H20C	109.5
H17D—O17—H17E	103 (3)	O3—C21—O2	124.5 (4)
O1—C1—C14	111.8 (3)	O3—C21—C22	117.7 (5)
O1—C1—C15	106.5 (3)	O2—C21—C22	117.7 (5)
C14—C1—C15	110.6 (3)	O3—C21—C22'	136.0 (6)
O1—C1—C2	103.7 (3)	O2—C21—C22'	99.4 (6)
C14—C1—C2	111.6 (3)	C22—C21—C22'	18.4 (4)
C15—C1—C2	112.4 (3)	C23—C22—C27	120.0
O2—C2—C3	108.0 (3)	C23—C22—C21	121.4 (4)
O2—C2—C1	103.5 (3)	C27—C22—C21	118.6 (4)
C3—C2—C1	118.6 (3)	C22—C23—C24	120.0
O2—C2—H2	108.8	C22—C23—H23	120.0
C3—C2—H2	108.8	C24—C23—H23	120.0
C1—C2—H2	108.8	F1—C24—C25	114.2 (7)
C4—C3—C2	112.4 (3)	F1—C24—C23	124.8 (7)
C4—C3—C8	110.9 (3)	C25—C24—C23	120.0
C2—C3—C8	115.3 (3)	C26—C25—C24	120.0
C4—C3—H3	105.8	C26—C25—H25	120.0
C2—C3—H3	105.8	C24—C25—H25	120.0
C8—C3—H3	105.8	C27—C26—C25	120.0
O4—C4—C5	113.0 (3)	C27—C26—H26	120.0
O4—C4—C18	110.5 (3)	C25—C26—H26	120.0
C5—C4—C18	83.9 (3)	C26—C27—C22	120.0
O4—C4—C3	107.9 (3)	C26—C27—H27	120.0
C5—C4—C3	120.5 (3)	C22—C27—H27	120.0
C18—C4—C3	119.4 (3)	C23'—C22'—C27'	120.0
O6—C5—C6	113.6 (3)	C23'—C22'—C21	109.8 (7)
O6—C5—C4	92.0 (3)	C27'—C22'—C21	129.3 (7)
C6—C5—C4	119.9 (3)	C24'—C23'—C22'	120.0
O6—C5—H5	110.0	C24'—C23'—H23'	120.0
C6—C5—H5	110.0	C22'—C23'—H23'	120.0
C4—C5—H5	110.0	F1'—C24'—C23'	142.2 (8)
C5—C6—C7	112.6 (3)	F1'—C24'—C25'	97.5 (8)
C5—C6—H6A	109.1	C23'—C24'—C25'	120.0
C7—C6—H6A	109.1	C26'—C25'—C24'	120.0
C5—C6—H6B	109.1	C26'—C25'—H25'	120.0
C7—C6—H6B	109.1	C24'—C25'—H25'	120.0
H6A—C6—H6B	107.8	C27'—C26'—C25'	120.0
O7—C7—C6	107.4 (3)	C27'—C26'—H26'	120.0
O7—C7—C8	107.9 (3)	C25'—C26'—H26'	120.0
C6—C7—C8	115.0 (3)	C26'—C27'—C22'	120.0
O7—C7—H7	108.8	C26'—C27'—H27'	120.0
C6—C7—H7	108.8	C22'—C27'—H27'	120.0
C8—C7—H7	108.8	O5—C28—O4	124.2 (4)
C19—C8—C7	112.5 (3)	O5—C28—C29	125.1 (4)
C19—C8—C9	107.0 (3)	O4—C28—C29	110.7 (4)
C7—C8—C9	104.5 (3)	C28—C29—H29A	109.5
C19—C8—C3	113.0 (3)	C28—C29—H29B	109.5
C7—C8—C3	103.7 (3)	H29A—C29—H29B	109.5
C9—C8—C3	115.9 (3)	C28—C29—H29C	109.5
O10—C9—C10	119.6 (3)	H29A—C29—H29C	109.5
O10—C9—C8	119.4 (3)	H29B—C29—H29C	109.5
C10—C9—C8	120.3 (3)	O8—C30—O7	127.4 (4)
O11—C10—C11	110.5 (3)	O8—C30—O9	125.9 (4)
O11—C10—C9	108.6 (3)	O7—C30—O9	106.7 (3)
C11—C10—C9	114.1 (3)	O9—C31—C32	108.2 (3)
O11—C10—H10	107.8	O9—C31—H31A	110.1
C11—C10—H10	107.8	C32—C31—H31A	110.1
C9—C10—H10	107.8	O9—C31—H31B	110.1
C12—C11—C10	119.5 (3)	C32—C31—H31B	110.1
C12—C11—C15	119.1 (3)	H31A—C31—H31B	108.4
C10—C11—C15	120.7 (3)	C31—C32—Cl2	107.2 (3)
C11—C12—C20	124.6 (4)	C31—C32—Cl3	110.9 (3)
C11—C12—C13	119.4 (4)	Cl2—C32—Cl3	110.0 (2)
C20—C12—C13	115.9 (3)	C31—C32—Cl1	110.3 (3)
O14—C13—C12	112.5 (4)	Cl2—C32—Cl1	109.7 (2)
O14—C13—C14	106.8 (3)	Cl3—C32—Cl1	108.8 (2)
C12—C13—C14	112.1 (3)	O12—C33—O11	127.7 (4)
O14—C13—H13	108.5	O12—C33—O13	125.2 (4)
C12—C13—H13	108.5	O11—C33—O13	107.1 (4)
C14—C13—H13	108.5	O13—C34—C35	108.0 (4)
C13—C14—C1	118.1 (3)	O13—C34—H34A	110.1
C13—C14—H14A	107.8	C35—C34—H34A	110.1
C1—C14—H14A	107.8	O13—C34—H34B	110.1
C13—C14—H14B	107.8	C35—C34—H34B	110.1
C1—C14—H14B	107.8	H34A—C34—H34B	108.4
H14A—C14—H14B	107.1	C34—C35—Cl6	107.5 (3)
C11—C15—C17	115.9 (3)	C34—C35—Cl4	112.0 (3)
C11—C15—C16	109.9 (3)	Cl6—C35—Cl4	109.0 (3)
C17—C15—C16	104.3 (3)	C34—C35—Cl5	110.1 (3)
C11—C15—C1	106.0 (3)	Cl6—C35—Cl5	109.3 (3)
C17—C15—C1	110.8 (3)	Cl4—C35—Cl5	108.9 (3)
C16—C15—C1	110.1 (3)	C37—C36—H36A	109.5
C15—C16—H16A	109.5	C37—C36—H36B	109.5
C15—C16—H16B	109.5	H36A—C36—H36B	109.5
H16A—C16—H16B	109.5	C37—C36—H36C	109.5
C15—C16—H16C	109.5	H36A—C36—H36C	109.5
H16A—C16—H16C	109.5	H36B—C36—H36C	109.5
H16B—C16—H16C	109.5	O15—C37—O16	116.5 (9)
C15—C17—H17A	109.5	O15—C37—C36	132.5 (9)
C15—C17—H17B	109.5	O16—C37—C36	111.0 (7)
H17A—C17—H17B	109.5	C39—C38—O16	109.7 (6)
C15—C17—H17C	109.5	C39—C38—H38A	109.7
H17A—C17—H17C	109.5	O16—C38—H38A	109.7
H17B—C17—H17C	109.5	C39—C38—H38B	109.7
O6—C18—C4	92.3 (3)	O16—C38—H38B	109.7
O6—C18—H18A	113.2	H38A—C38—H38B	108.2
C4—C18—H18A	113.2	C38—C39—H39A	109.5
O6—C18—H18B	113.2	C38—C39—H39B	109.5
C4—C18—H18B	113.2	H39A—C39—H39B	109.5
H18A—C18—H18B	110.6	C38—C39—H39C	109.5
C8—C19—H19A	109.5	H39A—C39—H39C	109.5
C8—C19—H19B	109.5	H39B—C39—H39C	109.5
			
C21—O2—C2—C3	128.3 (3)	C12—C11—C15—C17	175.8 (3)
C21—O2—C2—C1	−105.2 (3)	C10—C11—C15—C17	4.9 (5)
O1—C1—C2—O2	60.6 (3)	C12—C11—C15—C16	−66.3 (4)
C14—C1—C2—O2	−59.9 (4)	C10—C11—C15—C16	122.8 (4)
C15—C1—C2—O2	175.2 (3)	C12—C11—C15—C1	52.6 (4)
O1—C1—C2—C3	−179.9 (3)	C10—C11—C15—C1	−118.3 (3)
C14—C1—C2—C3	59.6 (4)	O1—C1—C15—C11	−177.1 (3)
C15—C1—C2—C3	−65.3 (4)	C14—C1—C15—C11	−55.4 (4)
O2—C2—C3—C4	−11.9 (4)	C2—C1—C15—C11	70.0 (4)
C1—C2—C3—C4	−129.0 (3)	O1—C1—C15—C17	56.6 (4)
O2—C2—C3—C8	−140.3 (3)	C14—C1—C15—C17	178.2 (3)
C1—C2—C3—C8	102.6 (4)	C2—C1—C15—C17	−56.4 (4)
C28—O4—C4—C5	−56.2 (4)	O1—C1—C15—C16	−58.3 (4)
C28—O4—C4—C18	−148.2 (3)	C14—C1—C15—C16	63.4 (4)
C28—O4—C4—C3	79.6 (4)	C2—C1—C15—C16	−171.2 (3)
C2—C3—C4—O4	74.7 (4)	C5—O6—C18—C4	−7.2 (3)
C8—C3—C4—O4	−154.6 (3)	O4—C4—C18—O6	119.2 (3)
C2—C3—C4—C5	−153.4 (3)	C5—C4—C18—O6	6.9 (3)
C8—C3—C4—C5	−22.7 (4)	C3—C4—C18—O6	−114.8 (3)
C2—C3—C4—C18	−52.4 (4)	C2—O2—C21—O3	−4.2 (6)
C8—C3—C4—C18	78.3 (4)	C2—O2—C21—C22	179.4 (4)
C18—O6—C5—C6	131.3 (3)	C2—O2—C21—C22'	177.5 (5)
C18—O6—C5—C4	7.3 (3)	O3—C21—C22—C23	15.6 (7)
O4—C4—C5—O6	−116.5 (3)	O2—C21—C22—C23	−167.8 (4)
C18—C4—C5—O6	−6.8 (3)	C22'—C21—C22—C23	−162 (3)
C3—C4—C5—O6	113.9 (3)	O3—C21—C22—C27	−165.1 (5)
O4—C4—C5—C6	124.7 (4)	O2—C21—C22—C27	11.5 (7)
C18—C4—C5—C6	−125.7 (4)	C22'—C21—C22—C27	18 (2)
C3—C4—C5—C6	−5.0 (5)	C27—C22—C23—C24	0.0
O6—C5—C6—C7	−111.6 (4)	C21—C22—C23—C24	179.2 (7)
C4—C5—C6—C7	−4.4 (5)	C22—C23—C24—F1	168.3 (10)
C30—O7—C7—C6	94.0 (4)	C22—C23—C24—C25	0.0
C30—O7—C7—C8	−141.5 (3)	F1—C24—C25—C26	−169.4 (9)
C5—C6—C7—O7	164.3 (3)	C23—C24—C25—C26	0.0
C5—C6—C7—C8	44.2 (4)	C24—C25—C26—C27	0.0
O7—C7—C8—C19	−68.0 (4)	C25—C26—C27—C22	0.0
C6—C7—C8—C19	51.8 (4)	C23—C22—C27—C26	0.0
O7—C7—C8—C9	47.7 (3)	C21—C22—C27—C26	−179.3 (7)
C6—C7—C8—C9	167.5 (3)	O3—C21—C22'—C23'	2.6 (11)
O7—C7—C8—C3	169.6 (3)	O2—C21—C22'—C23'	−179.5 (5)
C6—C7—C8—C3	−70.6 (4)	C22—C21—C22'—C23'	5.9 (19)
C4—C3—C8—C19	−66.2 (4)	O3—C21—C22'—C27'	−166.8 (7)
C2—C3—C8—C19	62.9 (4)	O2—C21—C22'—C27'	11.2 (9)
C4—C3—C8—C7	55.9 (3)	C22—C21—C22'—C27'	−163 (3)
C2—C3—C8—C7	−175.0 (3)	C27'—C22'—C23'—C24'	0.0
C4—C3—C8—C9	169.8 (3)	C21—C22'—C23'—C24'	−170.5 (9)
C2—C3—C8—C9	−61.1 (4)	C22'—C23'—C24'—F1'	172 (2)
C19—C8—C9—O10	18.4 (4)	C22'—C23'—C24'—C25'	0.0
C7—C8—C9—O10	−101.1 (4)	F1'—C24'—C25'—C26'	−175.2 (13)
C3—C8—C9—O10	145.4 (3)	C23'—C24'—C25'—C26'	0.0
C19—C8—C9—C10	−170.8 (3)	C24'—C25'—C26'—C27'	0.0
C7—C8—C9—C10	69.7 (4)	C25'—C26'—C27'—C22'	0.0
C3—C8—C9—C10	−43.8 (4)	C23'—C22'—C27'—C26'	0.0
C33—O11—C10—C11	−158.6 (3)	C21—C22'—C27'—C26'	168.4 (11)
C33—O11—C10—C9	75.6 (4)	C4—O4—C28—O5	−2.3 (6)
O10—C9—C10—O11	−5.4 (5)	C4—O4—C28—C29	178.2 (3)
C8—C9—C10—O11	−176.2 (3)	C7—O7—C30—O8	2.3 (6)
O10—C9—C10—C11	−129.2 (4)	C7—O7—C30—O9	−177.5 (3)
C8—C9—C10—C11	60.0 (4)	C31—O9—C30—O8	9.1 (6)
O11—C10—C11—C12	115.7 (4)	C31—O9—C30—O7	−171.1 (3)
C9—C10—C11—C12	−121.6 (4)	C30—O9—C31—C32	−174.4 (3)
O11—C10—C11—C15	−73.5 (4)	O9—C31—C32—Cl2	180.0 (3)
C9—C10—C11—C15	49.2 (4)	O9—C31—C32—Cl3	59.9 (4)
C10—C11—C12—C20	−11.8 (5)	O9—C31—C32—Cl1	−60.7 (4)
C15—C11—C12—C20	177.3 (3)	C10—O11—C33—O12	9.0 (6)
C10—C11—C12—C13	165.0 (3)	C10—O11—C33—O13	−171.1 (3)
C15—C11—C12—C13	−6.0 (5)	C34—O13—C33—O12	0.0 (6)
C11—C12—C13—O14	−155.7 (3)	C34—O13—C33—O11	−179.8 (3)
C20—C12—C13—O14	21.3 (5)	C33—O13—C34—C35	170.1 (4)
C11—C12—C13—C14	−35.3 (5)	O13—C34—C35—Cl6	178.6 (3)
C20—C12—C13—C14	141.6 (4)	O13—C34—C35—Cl4	58.9 (4)
O14—C13—C14—C1	151.0 (3)	O13—C34—C35—Cl5	−62.5 (5)
C12—C13—C14—C1	27.4 (5)	C38—O16—C37—O15	3.0 (11)
O1—C1—C14—C13	136.6 (4)	C38—O16—C37—C36	−177.4 (8)
C15—C1—C14—C13	18.1 (5)	C37—O16—C38—C39	171.4 (8)
C2—C1—C14—C13	−107.8 (4)		

### Hydrogen-bond geometry (Å, °)

**Table:**

*D*—H···*A*	*D*—H	H···*A*	*D*···*A*	*D*—H···*A*
O17—H17E···Cl1^i^	0.89 (2)	2.85 (6)	3.582 (5)	141 (6)
O17—H17E···O9^i^	0.89 (2)	2.48 (7)	3.241 (6)	143 (9)
O14—H14···O17	0.83 (2)	2.00 (2)	2.791 (6)	161 (4)
O1—H1···O15	0.82 (2)	2.01 (3)	2.786 (6)	158 (5)
O17—H17D···O1^ii^	0.91 (2)	2.25 (4)	3.085 (6)	152 (6)

Symmetry codes: (i) −*x*+1, *y*+1/2, −*z*+3/2; (ii) *x*+1/2, −*y*+1/2, −*z*+1.

## References

[bb1] Bruker (2001). *SAINT*, *SMART* and *SADABS* Bruker AXS Inc., Madison, Wisconsin, USA.

[bb2] Flack, H. D. (1983). *Acta Cryst.* A**39**, 876–881.

[bb3] Kingston, D. G. I., Hawkins, D. R. & Ovington, L. (1982). *J. Nat. Prod.* **45**, 466–470. 10.1021/np50022a019 7130988

[bb4] Lu, H.-F., Sun, X., Xu, L., Lou, L.-G. & Lin, G.-Q. (2009). *Eur. J. Med. Chem.* **44**, 482–491. 10.1016/j.ejmech.2008.04.004 18524425

[bb5] Sheldrick, G. M. (2008). *Acta Cryst.* A**64**, 112–122. 10.1107/S0108767307043930 18156677

